# Immune system and tumor microenvironment in early-stage breast cancer: different mechanisms for early recurrence after mastectomy and chemotherapy on ductal and lobular types

**DOI:** 10.12688/f1000research.134302.2

**Published:** 2023-09-29

**Authors:** Andreas Andrianto, I Ketut Sudiana, Desak Gede Agung Suprabawati, Hari Basuki Notobroto

**Affiliations:** 1 Doctoral Program of Medical Science, Faculty of Medicine, Universitas Airlangga, Surabaya, East Java, 60132, Indonesia; 2 Department of Pathology Anatomy, Faculty of Medicine, Universitas Airlangga, Surabaya, East Java, 60132, Indonesia; 3 Division of Oncology, Department of Surgery, Faculty of Medicine, Universitas Airlangga, Surabaya, East Java, 60132, Indonesia; 4 Department of Biostatistics and Population, Faculty of Public Health, Universitas Airlangga, Surabaya, East Java, 60132, Indonesia

**Keywords:** Ductal Carcinoma, Lobular Carcinoma, Local Recurrence, Early Breast Carcinoma

## Abstract

**Background:** The most common type of breast cancer is the ductal type (IDC), followed by lobular type (ILC). Surgery is the main therapy for early-stage breast cancer. Adjuvant chemotherapy might be given to those at high risk of recurrence. Recurrence is still possible after mastectomy and chemotherapy and most often occurs in the first two years. We aimed to determine the mechanisms in early local recurrence in both types.

**Methods:** We used an observational method with a cross-sectional study design. The samples were patients with early-stage IDC and ILC, who underwent modified radical mastectomy (MRM) and got adjuvant chemotherapy with taxan and anthracycline base, and experienced recurrence in the first two years after surgery. The materials in this study were paraffin blocks from surgical specimens; we examined vimentin, α-SMA and MMP1, PDGF and CD95 by immunohistochemistry (IHC). Data analysis was done using OpenEpi 3.0.1 and EZR. We used pathway analysis with linear regression.

**Results:** There were 25 samples with local recurrence and 25 samples without recurrence in the ductal type group. The lobular type group consisted of six subjects without recurrence and seven with recurrence. There were significant differences in the expression of vimentin (p=0.000 and 0.021, respectively), PDGF (p=0.000 and 0.002) and CD95 (p=0.000 and 0.045) in ductal and lobular cancer types, respectively. MMP1 (p=0.000) and α-SMA (p=0.000) only showed a significant difference in the ductal type. The pathway analysis showed that in the ductal type, the mechanism of recurrence was enabled by two factors: α-SMA and CD95. Meanwhile, for the lobular type, the recurrence mechanism was through the CD95 pathway.

**Conclusions:** Local recurrence in early-stage IDC and ILC had different mechanisms.  These findings are expected to make cancer treatment in both types more focused and efficient.

## Introduction

Metastasis in breast cancer occurs when cancer cells can migrate and survive against the body's defense mechanisms. ^1^ These cancer cells must be able to change the structure of their cytoskeleton and the surrounding extracellular matrix to support the migration process and avoid the apoptotic process to survive and metastasize, by producing proteins to protect themselves and changing signaling pathways so that the apoptotic process does not occur. ^2^

Breast cancer is still a major health problem both in the world and in Indonesia. In Indonesia, the highest incidence of cancer in women is breast cancer, which is 42.1 per 100,000 population, with an average death rate of 17 per 100,000 population. ^3^ The two most common histological types are 80% invasive ductal carcinoma (IDC), followed by 15% invasive lobular carcinoma (ILC). ^4^ Which type shows the better prognosis is still controversial. ^5^ Local recurrence and distant metastases after surgery are more common in the ILC type. ^6^ The high number of metastases in the ILC type is due to its nature, which tends to be multicentric and can invade the stroma without causing excessive stromal reactions, making it difficult for anatomic pathologists to determine the radicality of surgery. ^7^^,^^8^

Two surgical procedures that are often performed on breast cancer are breast conserving surgery (BCS) and modified radical mastectomy (MRM). ^9^^,^^10^ MRM is a procedure for removing the tumor and the skin over the tumor, all breast glands, accompanied by axillary dissection which is carried out simultaneously. ^11^ After MRM procedure, recurrence and metastases are still common even though the entire breast has been removed, even after adjuvant chemotherapy. ^12^

The most critical time for local recurrence in both types of histology is the first two years after the primary surgery. ^13^^,^^14^ The choice of management of local recurrence after a BCS procedure is re-excision or salvage mastectomy, ^13^ but the decision on which procedure to choose is still being debated because of the high rate of post-re-excision metastases. ^15^ Until now, the management of local recurrence after MRM has not been standardized, *i.e.,* whether re-excision can be carried out or treated in a setting like stage IV. ^13^^,^^16^

Surgery for residual breast cancer is thought to cause shedding of tumor cells into the bloodstream and lymph nodes. ^17^ Surgery will also suppress cellular mediated immunity (CMI) so that cancer cells are more aggressive and go through metastasis more easily. ^18^ Surgery for breast cancer that has experienced metastases can also reduce the distant anti-angiogenic effect so that it can trigger new metastatic foci in distant organs. ^17^^,^^19^ Surgery and chemotherapy can increase the formation of reactive oxygen species (ROS), which in turn can protect cancer cells from apoptosis through the “anti-ROS” mechanism by Nuclear Factor Kappa Beta (NF-kB). In addition, low ROS levels can turn cancer cells into cancer stem cells (CSC) via the twist signaling pathway. ^20^

To survive and metastasize, cancer cells are also capable of changing their nature to become immortal (stemness) and capable of invasion/migration. Through the epithelial to mesenchymal transition (EMT) mechanism, cancer cells can change from an epithelial phenotype to a more mobile mesenchymal phenotype. ^21^ One of the factors that makes cancer stem cells (CSCs) immortal is their ability to avoid apoptosis triggered by the body’s immune system, namely by increasing the expression of cluster of differentiation 95 (CD95) which has an apoptotic function and triggers cancer stemness. ^22^

The occurrence of EMT in cancer cells is indicated by the increased expression of mesenchymal markers: vimentin, n-cadherin, and fibronectin. There is also a decrease in the expression of epithelial markers such as: e-cadherin, cytokeratin, claudin. ^23^^,^^24^ Cancer cells that have undergone the EMT process could migrate and metastasize through a mechanism that can convert normal fibroblast cells into cancer associated fibroblasts (CAF) via the Platelet Derived Growth Factor (PDGF) pathway. ^25^ CAF alters the structure and shape of fibroblasts to better support the metastatic process. The formation of CAFs can be identified by the increased expression of α-SMA which correlates with EMT events. ^23^^,^^26^^,^^27^ In addition, CAF can issue signals to induce matrix metalloproteinase (MMP) formation to degrade the extra cellular matrix so that the path for cancer cells to migrate becomes easier. MMP is a proteolytic enzyme that regulates the microenvironment around cells and its expression is always increased in cancer. ^28^ There are more than 21 types of MMPs, and in breast cancer the MMP expression that increases is MMP1. ^29^

This study was conducted to explain the differences in local recurrence mechanisms of ductal and lobular invasive breast carcinoma after mastectomy and chemotherapy. This study compared the expression of vimentin, α-SMA, MMP1, PDGF and CD95 in the two types of breast cancer to assess which factors play a greater role in the process of local recurrence in each type of breast cancer.

### Ethical approval and consent to participate

Before study began, this research obtained ethical approval from the Research Ethics Committee of the Faculty of Medicine, State University of Jember with number 1.537/H25.1.11/KE/2021. The first author works as surgical oncologist at Dr. Koesnadi General Hospital (RSDK) Bondowoso, East Java, Indonesia. The research samples were taken at RSDK which belongs to the network of the Faculty of Medicine, Jember State University, where first author also teaches; therefore ethical approval was granted out by the ethics commission of Jember State University that acts as in-charge university. Currently, the first author is following a doctoral program at Airlangga University, East Java, Indonesia, so for the author's affiliation use Airlangga University, Surabaya. The co-authors are promotors and co-promotors for dissertation and they all are lecturers of Airlangga University. All patients whose sample took part in this study, after receiving an explanation about the study, signed a consent form to participate. If the patient died, the consent form is signed by their child or the patient’s next of kin.

## Methods

The research design used was analytic observational study with a case control study design. The subjects were patients with early-stage IDC and ILC (stages I and II), who had undergone MRM between January 2014 - December 2019 (five years) and received chemotherapy using a taxane and anthracycline base regimen, and experienced local recurrence within two years after surgery. All were operations performed by the first author. We obtained data from medical records in the surgical department of Dr H Koesnadi Bondowoso General Hospital, East Java, Indonesia (RSDK). The research subjects were divided into two groups for each type: a group with local recurrence and a group without local recurrence. The number of samples for IDC was 25 samples for each group. Determining the number of sample size based on the minimum number of samples needed for regression analysis The sampling technique for each ductal groups was simple random sampling. For ILC, number of samples was total subject who meet the inclusion and exclusion. This research was conducted at the RSUD Dr. Koesnadi General Hospital and Bhayangkara Hospital, East Java-Indonesia. The inclusion and exclusion criteria were as follows:

1. Inclusion criteria
  a. Patients with early stage lobular and ductal invasive breast cancer (early breast cancer) with locoregional recurrence after MRM and who have received chemotherapy for taxan and anthracycline base six times with an interval of three weeks (one series).
  b. There was an anatomic pathology examination report which includes:
    • Histological type
    • Grades
    • Radicality
    • Regional lymph node metastases
  c. There was a complete medical record of the patient including:
    • Patient's identity and age
    • Patient's hormonal status
    • Date of surgery and time of recurrence
    • Timing and type of chemotherapy regimen
2. Exclusion criteria
  a. The patient had received radiation therapy before.
  b. There was malignancy in other organs.
  c. The results of the pathological examination stated that the edges of the resection were not tumor-free, coincided or less than 1 cm.
  d. The paraffin block was damaged and could not be used.
  e. Paraffin blocks for surgical specimens could not be examined by immunohistochemistry because of poor fixation.

Block paraffin derived from the MRM operation specimen was cut to a thickness of 4 μm and then heated at 600°C for one hour. After that, deparaffinization was carried out three times using xylene solution for 3 minutes each. Furthermore, rehydration was carried out using 100%, 96%, and 70% ethanol. After rehydration, wash with water for 3 minutes. Peroxidase blockade was performed using 0.5% H _2_O _2_ for 30 minutes in methanol, then water cleansing for 5 minutes. Before and after peroxidase blockade, washing was carried out using pH 7.4 phosphate-buffer saline (PBS). Monoclonal antibodies used in this study were the Mouse anti-Human Monoclonal Antibody from MyBioSource: MBS475544 (vimentin), MBS2507725 (PDGF), MBS476188 (MMP1), MBS266274 (α-SMA) and MBS10754069 (CD95). The antibodies were optimized at a concentration of 1:600, diluted in phosphate-buffered saline with bovine serum albumin, and then incubated for 30 minutes at room temperature. The slides obtained were subjected to evaluation and analysis by quantifying the number of cells exhibiting a positive response to each specific antibody. This assessment was conducted using a light microscope set at a magnification of 400x. The calculation was conducted across ten fields of view, after which the average number of positive cells per field of view was determined. A statistical analysis was conducted in order to ascertain the disparities between the group that experienced recurrence and the group that did not experience recurrence. The statistical test employed in this study is the mean difference test. Prior to doing pathway analysis, it is customary to perform a normality test and homogeneity test, which are subsequently followed by a logistic regression test. The data processing program utilized is OpenEpi version 3.0, along with the free statistical software EZR, which is built upon R-command. The statistical value considered significant was p<0.05.

## Results

### Study characteristics

The research selected 50 patients with early-stage ductal type breast cancer and 13 patients with lobular type. The ductal type was divided into two groups: the first group comprised 25 patients who had local recurrence in the first two years and a control group of 25 patients who did not had local recurrence. In the lobular type group, the division was also carried out with the number of patients who had local recurrence, amounting to seven patients, and those who did not had local recurrence, comprising six patients ( Table 1 and Table 2).

**Table 1.** Characteristics of the research sample on the ductal type.

	Negative recurrence (n=25)	Positive recurrence (n=25)	p
**Age (mean)**	51.72	49.96	0.59
**Lymph node metastases**	3.04	3.92	0.527
**Grade**	1.96	2	0.166
**Hormonal status**			
Pre-menopause	12	14	0.5713
Menopause	13	11	

Statistical test from ILC also showed no significance diference based on on age, lymphnode metastasis, tuor grade and hormonal status on ductal type breast cancer ( Table 2).

**Table 2.** Characteristics of the research sample on the lobular type.

	Negative recurrence (n=6)	Positive recurrence (n=7)	p
**Age (mean)**	46.83	51.00	0.532
**Lymph nodes metastases**	1.33	3.00	0.07
**Grade**	1.86	2	0.631
**Hormonal status**			
Pre-menopause	3	5	0.428
Menopause	3	2	

### Data analysis

Before we carried out statistical tests, we tested for normality and homogeneity of data distribution. For data that were not normally distributed, the statistical test used was a non-parametric Mann-Whitney test, while for data which had a normal distribution, we used a parametric independent t test.

Based on Table 3, there was a significant difference in vimentin expression between the group that experienced recurrence and the group that did not experience recurrence, where for the ductal type p=0.000 and for the lobular type p=0.021.

**Table 3.** Statistical test results of vimentin expression for ductal and lobular types.

Invasive breast cancer type	Vimentin expression		p
	Non-recurrence	Recurrence	
Ductal	2.24±1.72	8.32±5.74	0.000 *
	1.50 (0.4-7.2)	6.00 (2.3-21.0)	
Lobular	1.43±1.50	8.16±5.75	0.021 **
	0.90 (0.0-4.0)	9.10 (1.9-18.5)	
p	0.168 *	0.909 *	

Description: data in mean SD, median, (Minimum-Maximum)±.

* Mann-Whitney test.

** Independent t-test.

According to the test results, there was a significant difference in PDGF expression between the group that experienced recurrence and the group that did not experience recurrence in both types of breast cancer, where for the ductal type p=0.000 and for the lobular type p=0.002 ( Table 4).

**Table 4.** Statistical test results of platelet-derived growth factor (PDGF) expression for ductal and lobular types.

Invasive breast cancer type	PDGF expression		p
	Non-recurrence	Recurrence	
Ductal	3.95±2.70	14.95±5.36	0.000 *
	3.20 (0.9-10.7)	15.60 (6.4-24.2)	
Lobular	1.62±1.58	6.96±3.01	0.002 **
	1.35 (0.0-4.4)	6.40 (4.0-11.3)	
p	0.030 *	0.001 **	

Description: data in Mean SD, Median, (Minimum-Maximum)±.

* Mann-Whitney test.

** Independent t-test.

According to the results of the tests performed, a significant difference in MMP1 expression was only found between the recurrence and non-recurrence groups in the ductal type group (p=0.000), while in the lobular type there was no significant difference in MMP1 expression in the non- recurrence group compared to the recurrence group (p=0.102) ( Table 5).

**Table 5.** Statistical test results of matrix metalloproteinase (MMP1) expression for ductal and lobular types.

Invasive breast cancer type	MMP1 expression		p
	Non-recurrence	Recurrence	
Ductal	1.48±1.13	3.24±1.77	0.000 *
	1.20 (0.3-4.6)	3.00 (1.1-7.3)	
Lobular	9.43±9.31	17.76±7.56	0.102 **
	7.65 (0.0-21.2)	19.80 (6.9-28.5)	
p	0.211 *	0.002 **	

Description: data in mean SD, median, (minimum-maximum)±.

* Mann-Whitney test.

** Independent t-test.

Based on the results for α-SMA expression, a significant difference was found in α-SMA expression in the ductal type (p=0.000) while in the lobular type there was no significant difference (p=0.063) ( Table 6).

**Table 6.** Statistical test results of expression α-SMA for ductal and lobular types.

Invasive breast cancer type	α-SMA expression		p
	Non-recurrence	Recurrence	
Ductal	3.08±1.37	5.59±1.85	0.000 *
	3.00 (0.4-5.4)	5.10 (3.2-12.8)	
Lobular	10.12±6.40	22.13±9.39	0.063 *
	13.70 (0.0-15.0)	23.90 (9.9-31.5)	
p	0.030 *	0.000 *	

Description: data in mean SD, median, (minimum-maximum)±.

* Mann-Whitney test.

According to the results of the tests conducted, there was a significant difference in CD95 expression between the group that experienced recurrence and the group that did not experience recurrence for both cancer types, where for the ductal type p=0.000 and for the lobular type (p=0.045) ( Table 7).

**Table 7.** Statistical test results of CD95 expression for ductal and lobular types.

Invasive breast cancer type	CD95 expression		p
	Non-recurrence	Recurrence	
Ductal	8.22±5.92	0.92±0.78	0.000 *
	6.20 (0.4-5.4)	0.60 (0.0-2.7)	
Lobular	13.17±6.80	1.53±0.69	0.045 *
	14.90 (0.0-18.7)	1.80 (0.4-2.4)	
p	0.140 *	0.064 *	

Description: data in mean SD, median, (minimum-maximum)±.

* Mann-Whitney test.

### Pathway analysis

**Figure 1.**
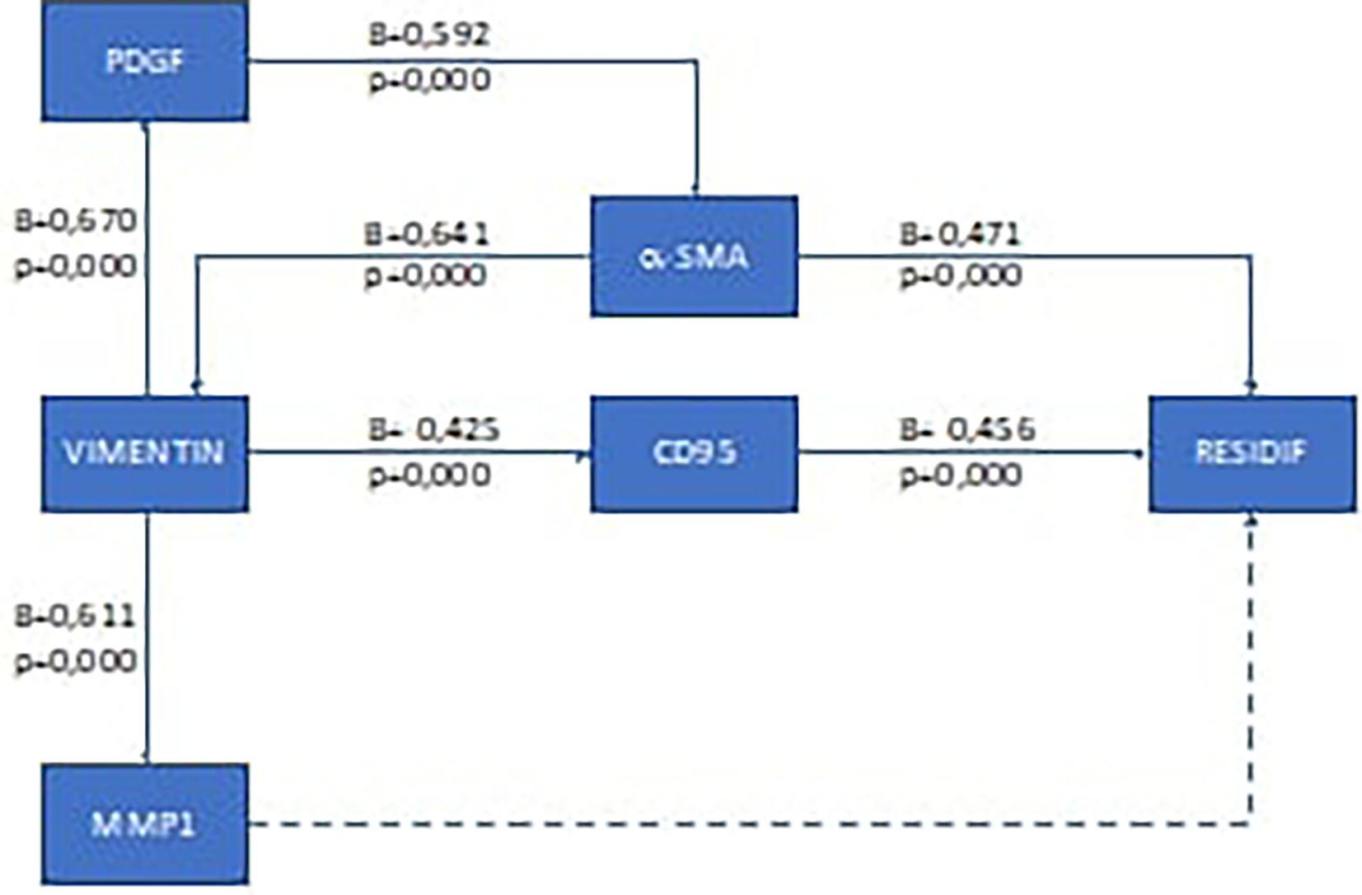
Results of recurrence mechanism pathway analysis in post-mastectomy ductal type breast cancer and adjuvant chemotherapy. Solid arrow: there is correlation; dotted arrow: there is no correlation.

**Figure 2.**
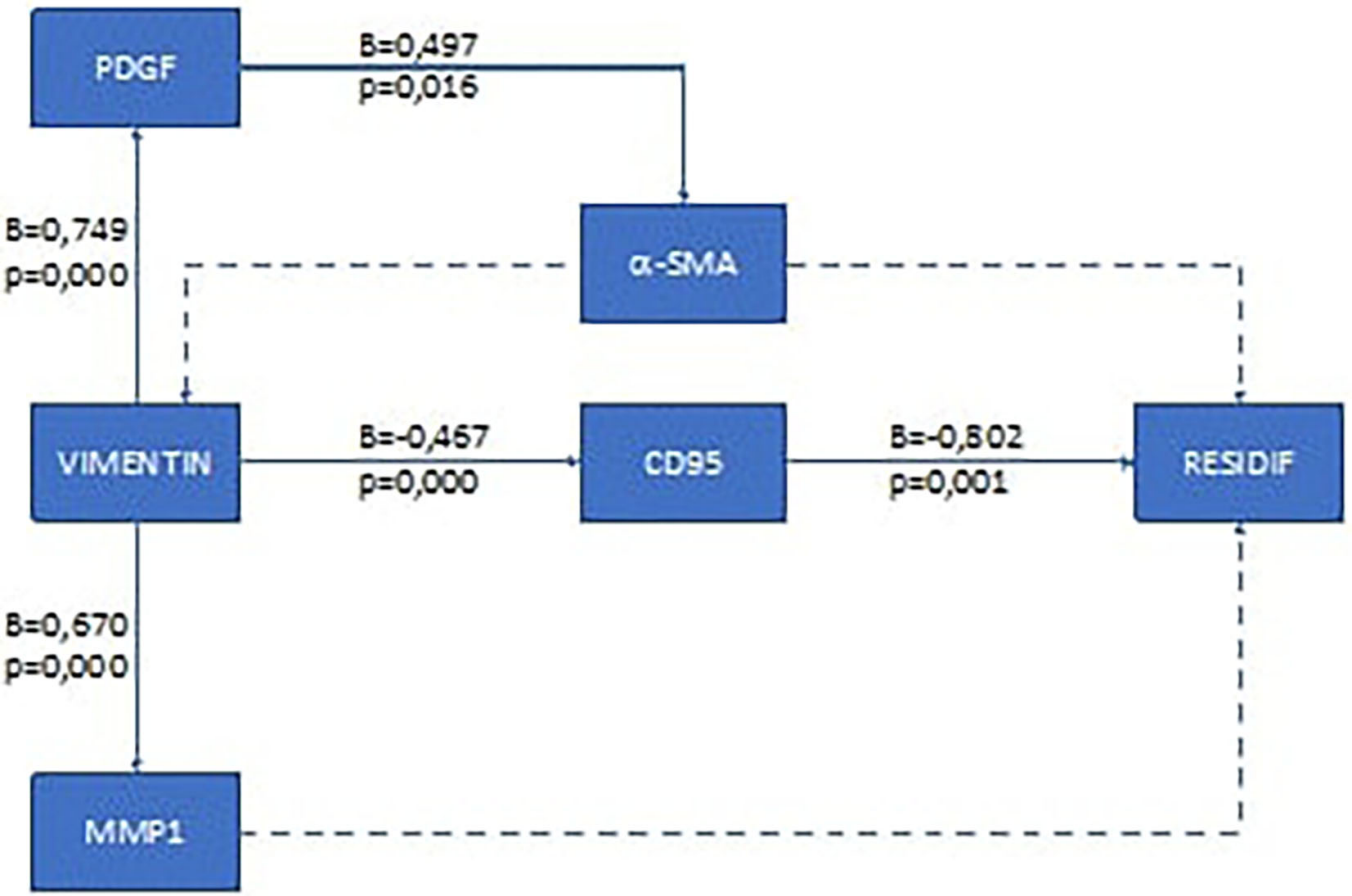
Results of recurrence mechanism pathway analysis in post-mastectomy lobular type breast cancer and adjuvant chemotherapy. Solid arrow: there is correlation; dotted arrow: there is no correlation.

To analyze the recurrence mechanism, we performed pathway analysis. The results of the pathway analysis for the ductal type breast cancer are presented in Figure 1, and for lobular type in Figure 2.

Based on these results, it was found that the recurrence mechanism in the early-stage ductal type breast cancer after mastectomy and chemotherapy was different from that of the lobular type. In the ductal type, the recurrence mechanism goes through two ways, namely through the pathway that affects the extra cellular structure of cancer cells (via the α-SMA pathway) and through the suppression of the body's immune cells (via the CD95 pathway). For the lobular type, the recurrence mechanism of this study was only through the CD95 pathway. The recurrence mechanism in the lobular type can elucidate why in the lobular type it is not very clear that changes in the extra cellular matrix in cancer cells will ultimately make it difficult to determine the outer edge of the tumor.

## Discussion

### Tumor microenvironment in breast cancer recurrence

Tumor microenvironment (TME) has long been a topic of research to determine the biological properties of tumor cells themselves and is one of the main factors in the process of tumor growth, metastases, and resistance to chemotherapy. ^30^ Many studies have mentioned that there is a two-way communication between tumor cells and TME which allows tumor cells to avoid the body's defense system, survive after chemotherapy and can spread to other places. ^31^

The main point in the process of recurrence and the occurrence of chemotherapy drug resistance is the occurrence of epithelial to mesenchymal transition (EMT), which can be detected using mesenchymal markers, including vimentin, N-cadherin, and fibronectin. Vimentin is the main component that makes up the cell skeleton (cytoskeleton). Also, vimentin acts as a protein forming the cell skeleton and plays a role in the process of cell motility. Vimentin expression is positive in cells that are actively dividing. Increased vimentin expression is associated with more aggressive tumor cell properties, increased ability to metastasize and poorer prognosis. ^32^ The actin structure of the cytoskeleton is a crucial element in the process of protrusion and cell migration, so that the intermediate cytoskeleton filament, especially vimentin, also plays a role in the process of adhesion and cell spread. ^33^ In addition, vimentin can protect cells against exposure to stress. ^34^

Abnormal vimentin expression is found in some types of cancer such as primary epithelial cancer or metastasis. Recent studies have found that vimentin also plays a role in the EMT process of breast cancer, resulting in a decrease in genes associated with invasion and such basal phenotypes. ^35^ Excessive expression of this compound correlates with poor prognosis in breast cancer patients. In addition, high vimentin levels are significantly associated with the spread and survival of breast cancer cells, allowing recurrence cancer cases. ^36^ This is in line with research by Yamashita in 2013, who stated that vimentin levels are associated with poor prognosis in recurrence breast cancer. ^37^ In addition, research conducted by Winter in 2021 showed an increase in vimentin activating the AKT pathway, which plays a role in increased breast cancer cell proliferation and invasion. ^38^

Based on the results of statistical tests conducted in this study, there were significant differences in vimentin expression between groups that had recurrence events and those who did not in the ductal type (p=0.000) and lobular type groups (p=0.021). This result is in line with research conducted by Wang, 2020, who found that vimentin overexpression was found in ductal type breast cancer cells. ^39^ This is due to the synergy between vimentin and LAP3, where LAP3 expression can increase vimentin expression. ^39^ In addition, the relationship between the two can also be significant because vimentin plays an important role in promoting the migration and invasion of breast cancer cells by LAP3. ^40^ Research conducted by Vora, 2009 also showed similar results, namely recurrence breast cancer patients having higher vimentin levels compared to non-recurrence breast cancer, both in lobular and ductal breast cancer types. ^41^ A study conducted by Rodrigez stated that vimentin expression in non-basal-like tumors was lower than that in basal-like tumors (p<0.001). Basal-like tumors correlate with poor prognosis and tend to recur, and vimentin expression in tumor cells correlates with recurrence. ^42^ Different result were obtained in a study conducted by Seshadri in 1996. This study stated that there is no significant association between vimentin expression and the risk of recurrence or death from breast cancer. In the same study, the authors also explained that vimentin plays a greater role only in tumors with negative hormone receptors. ^43^

In our study, there was a significant difference in MMP1 expression between the non-recurrence group and the recurrence group in the ductal type (p=0.000), while for the lobular type there was no significant difference (p=0.102). According to a study conducted by Del Caszar, increased expression of MMP1 is obtained especially in ductal type breast cancer when compared to other types of breast cancer (mucinous and lobular). ^44^ A study conducted by Shen *et al.* also mentioned that increased MMP1 expression in invasive breast cancer is correlated with the occurrence of resistance to chemotherapy drugs (multi-drug resistance). ^45^ In addition to causing chemoresistance, increased MMP1 expression is also correlated with the occurrence of resistance to hormonal therapy. ^46^ Another study also stated that increased MMP1 expression in breast cancer is correlated with recurrence and metastasis so that MMP1 can be used as a prognostic factor in breast cancer. ^47^

At the time of EMT in breast cancer, cancer cells induce increased PDGF expression through the TGF-β pathway. In normal cells, TGF-β functions to control homeostasis, maintaining the body's defense system and plays a role in the wound healing process. When cells are in a pre-malignant state, TGF-β plays a role in suppressing tumor growth directly, for example through apoptosis activation or indirectly by controlling the stroma around the cells ( *e.g.*: suppressing the inflammatory process). When the cell has become malignant and EMT has occurred, the cell can deactivate the ability of TGF-β to suppress tumor growth, so that what becomes dominant is the function of TGF-β as a trigger for tumor progression. ^48^

Cancer cells that have gone through EMT will make TGF-β produce cytokines that are pro-tumorigenic, including ILEI (Interleukin-like EMT-inducer), HGF (hepatocyte growth factor), EGF (epidermal growth factor) and PDGF (platelet-derived growth factor). ^49^ PDGF is a pro-angiogenic factor that plays a role in several solid tumor growth processes by paracrine and autocrine means. The signal derived from PDGF will make the tumor cells become more aggressive (autocrine), stimulate angiogenesis (paracrine) and turn normal fibroblasts into cancer-associated fibroblasts (CAF). Furthermore, the CAF will activate transcription factors (among others: SNAIL, SLUG) which can convert epithelial progenitor cells into mesenchymal progenitor cells. All these processes will result in more cells experiencing EMT, and a “loop signaling” allows cancer cells to multiply and progress. ^50^

CAF is the most abundant component found in the tumor microenvironment. In malignancy, CAF will determine the progress of tumor cells through regulating nutrition for tumor cells, re-shaping the extracellular matrix so that cancer cells can invade more easily, suppressing the body's defense system so that cancer cells are not destroyed by immune cells, and regulating extra and intra-cellular signals so that cancer cells can survive chemotherapy. ^51^ CAF has several markers including: α-SMA, FAP, integrin β1/CD29; where α-SMA is the most widely used marker for CAF. ^52^

In this study, significant differences were found for PDGF expression between the non-recurrence group and the recurrence group, both for the ductal type (p=0.000) and the lobular group (p=0.002). These results are the same as studies conducted by Jansson which stated that PDGF expression is correlated with the occurrence of early recurrence in breast cancer. ^53^ Another study conducted by Chou stated that breast cancer patients who get taxane chemotherapy tend to experience chemoresistance if they get over expression of PDGF. ^54^ Another study stated that if PDGF expression in breast cancer patients is inhibited, it increases the effectiveness of hormonal therapy in patients with positive hormonal receptors. ^55^

The expression α-SMA in this study showed that for ductal type breast cancer there was a significant difference between the non-recurrence group and the recurrence group (p=0.000). These results are the same as research by Bonneau which stated that α-SMA (CAF) expression correlates with recurrence and metastasis in luminal (ductal) type breast cancer. ^56^ In lobular breast cancer, α-SMA expression was not significantly different between the non-recurrence group and the recurrence group (p=0.063). These results were found since in lobular type breast cancer, there are no severe TME changes compared to the ductal type. ^44^ Recurrence that occurs in lobular type breast cancer often occurs due to a non-radical margin of operation because changes in the structure of the TME are not very clear, making it difficult for surgeons and pathologists to determine the outer boundary of the tumor. ^57^

### Immunity escape in breast cancer recurrence

Breast cancer cells that are still present when the patient undergoes therapy (surgery, chemotherapy, or radiotherapy) is in a dormant condition. While in dormant conditions, cancer cells adjust to new micro-environment conditions, try to survive chemotherapy and radiation, and adapt to avoid the body's defense mechanisms. ^58^

An important phase for cancer cells to come out of a dormant situation is when the breast cancer cells can change from an epithelial phenotype to mesenchymal phenotype (EMT). The EMT process makes cancer cells convert pro-apoptotic factors into non-apoptotic, so that the cells become immortal and have cancer stemness properties. When cancer cells are already in such a condition, they will be more resistant to multi-drug chemotherapy, more aggressive and likely to recur. ^59^

One of the pro-apoptotic factors that play an important role in controlling cancer cell growth is CD95. Activation of CD95 by cancer cells activates the death-inducing signaling complex (DISC) via the Fas-associated protein with death domain (FADD), caspase-8 and caspase-10 pathways. ^60^ When EMT occurs or if it is continuously stimulated, CD95 can change its nature from pro-apoptotic factor to non-apoptotic factor. ^61^ EMT and chronic stimulation of CD95 will make cancer cells secrete interferon type I (IFNα or IFNβ) which will interact with its receptors (IFNAR1 and IFNAR2). This interaction activates the signal transducer and activator of transcription 1 (STAT1) and causes STAT1-promoting cancer stemness. ^62^

The results of this study showed that there was a significant difference in CD95 expression between the group that experienced recurrence and did not experience recurrence in the Ductal type (p=0.000) and lobular type (p=0.045). Similar results were also mentioned by Pellegrino, reporting that CD95 expression is one of the risk factors for breast cancer recurrence. ^63^

### Recurrence mechanism in ductal and lobular type breast cancer

**Local recurrence mechanism in ductal type breast cancer**

The results of pathway analysis in ductal type recurrence breast cancer in this study showed a significant influence between vimentin expression and MMP1 expression (p=0.000) and had a strong correlation between the two (β=0.611). These results are in line with research conducted by Stallings-Mann, 2012, on Rac1b cells, showing that vimentin induces an increase in MMP1 expression. ^64^

Vimentin expression also had an influence on PDGF expression (p=0.000) and had a strong correlation coefficient (β=0.670). This result is in accordance with the findings of research conducted by Paulin, 2022, which stated that the occurrence of EMT (with vimentin markers) affects several growth factors, including PDGF, through the binding of basic protein heterodimers, leucine-zipper (bZIP), either Jun (c-Jun, JunB, JunD), Fos (cFos, FosB, Fra1 and Fra2), ATF (ATF-1, ATF-2)/CREB, or homodimers from Jun/Jun. ^65^

PDGF in this study also showed a significant effect on α-SMA expression (p=0.000) with a moderate correlation (β=0.592). Similar results were also shown in previous research by Valgeirsdóttir, 1998. The study also added that activation of the PDGF receptor causes reorganization of vimentin through the associated fibroblast cancer pathway of which α-SMA is a marker. ^66^

The expression of α-SMA in this study had an influence on the occurrence of recurrence in ductal type breast cancer (p=0.000) with a moderate correlation (β=0.592). These results are in line with a study conducted by Bonneau which stated that CAF correlates with the occurrence of recurrence in early-stage ductal type breast cancer. ^56^ Another study conducted by Risom also stated that changes in the structure and composition of the stroma of cancer cells due to CAF activation causes cancer cells to become more aggressive and increase the risk of recurrence. ^67^

The occurrence of recurrence in this study was also influenced by EMT which resulted in the body's defense mechanism impairment. Pathway analysis in this study showed that EMT influenced CD95 (p=0.000) with a correlation value of β=0.592 (moderate correlation). Furthermore, CD95 expression significantly influenced the occurrence of recurrence (p=0.000). In a previous study, Guégan also mentioned the same thing, reporting that CD95 expression correlated with recurrence and resistance to chemotherapy in ductal type breast cancer. ^68^

**Local recurrence mechanism in lobular type breast cancer**

For lobular type breast cancer, in this study the occurrence of recurrence was influenced by the failure of the body's defense mechanism to destroy cancer cells. The TME in this study did not have a significant influence on the occurrence of recurrence. Research conducted by Van der Sangen concluded that TME in lobular type breast cancer affects the occurrence of recurrence if the surgery performed is not radical. ^69^

The EMT process that occurs in lobular breast cancer makes CD95 a significant non-apoptotic factor (p=0.000) and has a moderate correlation coefficient (β=0.467). These changes give cancer cells properties like those of cancer stem cells and make them immortal. ^62^ This is evidenced by this study, which shows that CD95 affects the occurrence of recurrence (p=0.001) and has a very strong correlation (β=0.802). The nature of cancer cells that become immortal will make the cancer cells resistant to chemotherapy and cause recurrence. These results are similar to a study by Wilson which stated that lobular type breast cancer is more resistant to chemotherapy when compared to ductal type breast cancer. ^70^

According on the results of observations and statistical analysis, it was found that the number of cells expressing vimentin, MMP1, and PDGF in ductal type cancers was different from the lobular type cases that underwent local recurrence after mastectomy and adjuvant chemotherapy. Meanwhile, we found no difference in α-SMA and CD95. Besides that, the local recurrence mechanism in the ductal type of early-stage breast cancer is different from that of the lobular type. In the ductal type, the mechanism of recurrence happens through two pathways: through pathways that affect the tumor microenvironment and through pathways that affect the body's defense mechanisms. Whereas for lobular breast cancer, the local recurrence mechanism is only through pathways that affect the body's defense mechanisms. Finally, author realize that breast cancer cases are very heterogeneous; the heterogenicities of the sample and the small number of samples were weakness in this study additional study that involves various centres could potentially yield benefits.

## Data Availability

Figshare: RAW Data for surgical specimens subjected to immunohistochemical examination using *monoclonal antibodies* against vimentin, α-SMA and MT-MMP1, PDGF and CD95, https://doi.org/10.6084/m9.figshare.22817513.v1. ^71^ Data are available under the terms of the Creative Commons Zero “No rights reserved” data waiver (CC0 1.0 Public domain dedication)
