# Supplementary material for: Immune system and tumor microenvironment in early-stage breast cancer: different mechanisms for early recurrence after mastectomy and chemotherapy on ductal and lobular types
Source: F1000Res. 2023 Sep 29;12:841. Originally published 2023 Jul 18. [Version 2] doi: 10.12688/f1000research.134302.2 (PMC10692586; doi:10.12688/f1000research.134302.2)
Supplement: Peer review history

# Reviewer report: Reviewer response for version 2

**DOI:** 10.5256/f1000research.156682.r210984

**Published:** 2023-12-01

**Authors:** Sumadi Lukman Anwar

**Affiliations:** 1 Division of Surgical Oncology Department of Surgery, RSUP Dr Sardjito / Faculty of Medicine, Public Health, and Nursing, Universitas Gadjah Mada, Yogyakarta, Indonesia

The authors have revised the manuscript according to the suggestions raised by the reviewers. However, it would be better for the authors to revise the grammar, syntax, and wording so that the manuscript can be easily understood by the readers.

Is the work clearly and accurately presented and does it cite the current literature?

Partly

If applicable, is the statistical analysis and its interpretation appropriate?

Partly

Are all the source data underlying the results available to ensure full reproducibility?

Partly

Is the study design appropriate and is the work technically sound?

Partly

Are the conclusions drawn adequately supported by the results?

No

Are sufficient details of methods and analysis provided to allow replication by others?

Partly

Reviewer Expertise:

NA

I confirm that I have read this submission and believe that I have an appropriate level of expertise to confirm that it is of an acceptable scientific standard.

# Reviewer report: Reviewer response for version 1

**DOI:** 10.5256/f1000research.147348.r205651

**Published:** 2023-09-26

**Authors:** Kristanto Yuli Yarso

**Affiliations:** 1 Oncology Surgery Division, Department of Surgery, Sebelas Maret University, Surakarta, Indonesia

1. Please explain abbreviations when first used: IDC, ILC, SMA, MMP1, PDGF.
2. When you chose cancer recurrence as entry point and followed retrospectively then obtained many predicting factors from MRM tissue - this should be put as case control design.
3. No need to discuss about BCS, since all your subjects were mastectomy patients.
4. No need to explain data distribution analysis since it is standard procedure, just present data in "mean" whenever it has normal distribution and "median" when it has no normal distribution.
5. More subjects would be more appropriate.

Is the work clearly and accurately presented and does it cite the current literature?

Partly

If applicable, is the statistical analysis and its interpretation appropriate?

Yes

Are all the source data underlying the results available to ensure full reproducibility?

Yes

Is the study design appropriate and is the work technically sound?

Partly

Are the conclusions drawn adequately supported by the results?

Partly

Are sufficient details of methods and analysis provided to allow replication by others?

Yes

Reviewer Expertise:

molecular biology, tumor microenvironment, breast cancer

I confirm that I have read this submission and believe that I have an appropriate level of expertise to confirm that it is of an acceptable scientific standard.

# Response

**Published:** 2023-09-26

**Authors:** Andreas Andrianto

**Affiliations:** Doctoral Program, Universitas Airlangga, Surabaya, East Java, Indonesia

I express my gratitude for dedicating your time to evaluate the content of my manuscript.

 1. The explanation of abbreviations in the main text has been revised to accommodate the restricted character limitation in the abstract.

 2. The study design has been revised in accordance with the recommendations provided by the reviewers.

 3. The explanation regarding BCS has been omitted.

 4. We appreciate your input, and we have made the necessary revisions.

 5. We have added it to the conclusions and suggestions section

# Reviewer report: Reviewer response for version 1

**DOI:** 10.5256/f1000research.147348.r205648

**Published:** 2023-09-22

**Authors:** Sumadi Lukman Anwar

**Affiliations:** 1 Division of Surgical Oncology Department of Surgery, RSUP Dr Sardjito / Faculty of Medicine, Public Health, and Nursing, Universitas Gadjah Mada, Yogyakarta, Indonesia

The study provides new insights in the different expression of some proteins between ductal and lobular breast carcinomas. However, there are some rooms for improvement:

- In the methods: please provide antibody clones used in the study and the company so that other researchers can repeat the experiments. The incubation time, temperature, and processing during the IHC should be provided. 
- Immunohistochemistry could be interpreted by pathologists. In this study, image analysis should be explained more details, calculation of the images, statistical analysis should be explained
- In the methods, pathway analysis should be explained in more detail, which software or enrichment analysis that has been used and how to develop the pathway.
- In the conclusion (abstract), the authors should explain more in the different mechanisms of lobular and ductal carcinomas and which cancer treatment that can be modified according to this study

Is the work clearly and accurately presented and does it cite the current literature?

Partly

If applicable, is the statistical analysis and its interpretation appropriate?

Partly

Are all the source data underlying the results available to ensure full reproducibility?

Partly

Is the study design appropriate and is the work technically sound?

Partly

Are the conclusions drawn adequately supported by the results?

No

Are sufficient details of methods and analysis provided to allow replication by others?

Partly

Reviewer Expertise:

Breast cancer, hnscc, molecular cancer

I confirm that I have read this submission and believe that I have an appropriate level of expertise to confirm that it is of an acceptable scientific standard, however I have significant reservations, as outlined above.

# Response

**Published:** 2023-09-23

**Authors:** Andreas Andrianto

**Affiliations:** Doctoral Program, Universitas Airlangga, Surabaya, East Java, Indonesia

**Immunohistochemistry (IHC) Staining and Assessment**

 Block paraffin derived from the MRM operation specimen was cut to a thickness of 4 µm and then heated at 600°C for one hour. After that, deparaffinization was carried out 3 times using xylene solution for 3 minutes each. Furthermore, rehydration was carried out using 100%, 96%, and 70% ethanol. After rehydration, wash with water for 3 minutes. Peroxidase blockade was performed using 0.5% H2O2 in thirty minutes of methanol, then water cleansing for 5 minutes. Before and after peroxidase blockade, washing was carried out using pH 7.4 Phosphate-buffer saline (PBS). Immunohistochemical staining was carried out on all tumor samples to identify vimentin, α-SMA, PDGF, MMP1, CD95 protein expression. The antibodies used for the study were Mouse anti-human Monoclonal Antibody from MyBioSource with the catalog number: MBS475544 vimentin), MBS2507725 (PDGF), MBS476188 (MMP1), MBS266274 (α-SMA ) and MBS10754069 (CD95). ). The antibodies were optimized at a concentration of 1:600, diluted in phosphate-buffered saline (PBS) with bovine serum albumin (BSA), and then incubated with the tumor sections for 30 minutes at room temperature.

 The slides obtained were subjected to evaluation and analysis by quantifying the number of cells exhibiting a positive response to each specific antibody. This assessment was conducted using a light microscope set at a magnification of 400x. The calculation was conducted across ten fields of view, after which the average number of positive cells per field of view was determined.

 A statistical analysis was conducted in order to ascertain the disparities between the group that experienced recurrence and the group that did not experience recurrence. The statistical test employed in this study is the mean difference test. Prior to doing pathway analysis, it is customary to perform a normality test and homogeneity test, which are subsequently followed by a logistic regression test. The data processing program utilized is OpenEpi version 3.0, along with the free statistical software EZR, which is built upon R-command. The statistical value that is considered significant is <0.05

**Conclusion**

 The processes of local recurrence in early-stage IDC and ILC exhibit distinct characteristics. The tumor microenvironment (TME) and the immune system are both critical factors in determining the likelihood of recurrence in IDC, while in ILC, the immune system plays a crucial role in determining the likelihood of recurrence. The results of this study indicate that there is merit in exploring the augmentation of the patient's immune system as a viable approach for the treatment of cancer.
